# Supplementary figures and images for: High-throughput 16S rRNA gene sequencing reveals that 6-hydroxydopamine affects gut microbial environment
Source: PLoS One. 2019 Aug 12;14(8):e0217194. doi: 10.1371/journal.pone.0217194 (PMC6690581; doi:10.1371/journal.pone.0217194)

**A**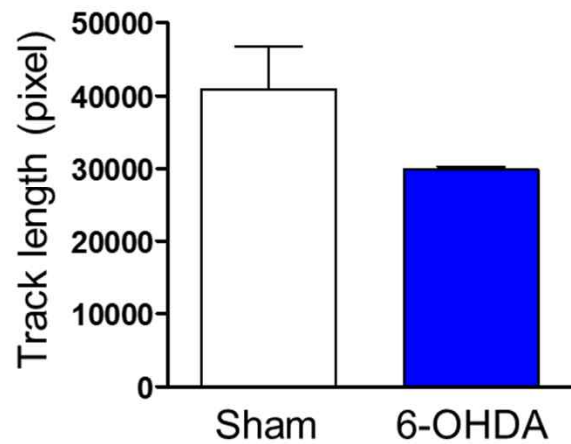**B**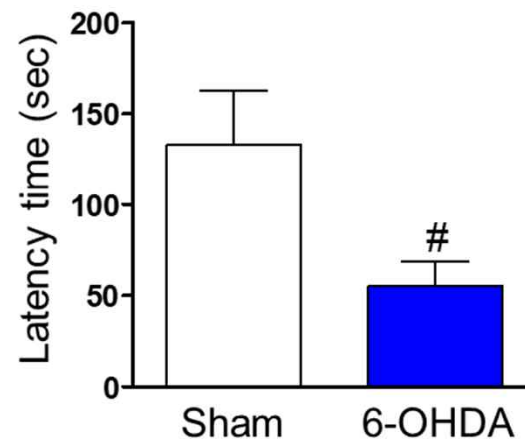**C**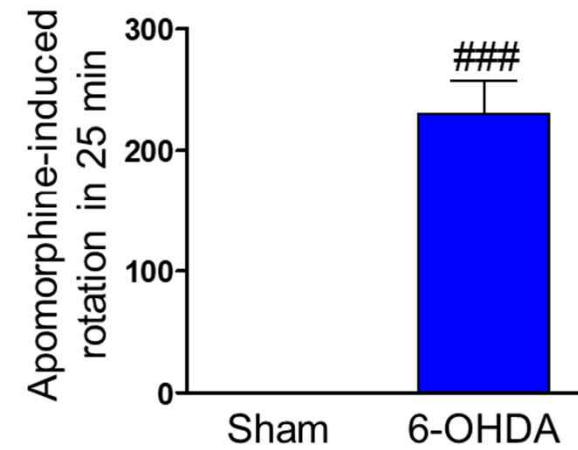**D**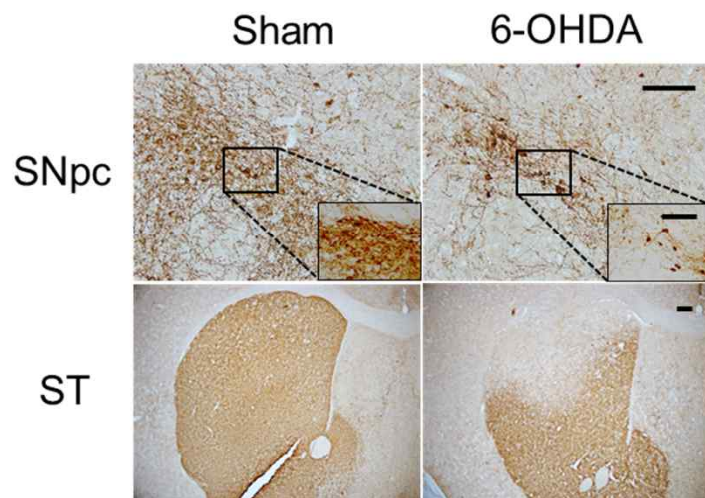**E**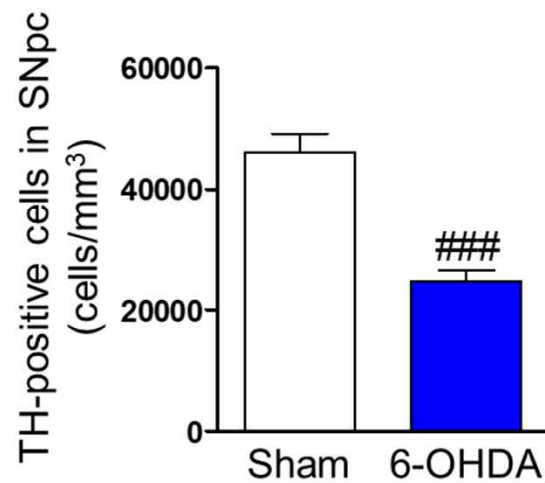**F**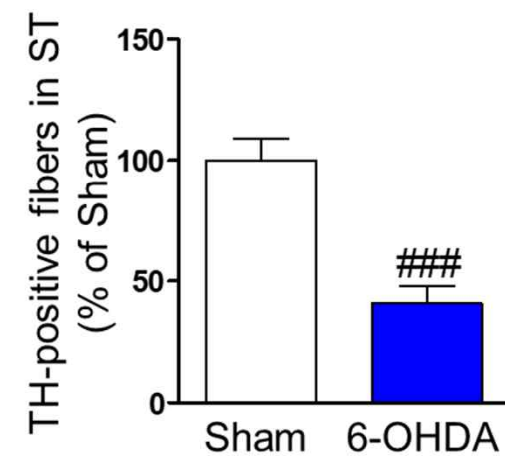

Supplement: S1 Fig — (A-C) open field test, rotarod test, and apomorphine-induced rotation test were performed to explore whether 6-OHDA induces motor deficits in mice, respectively. (D-F) immunohistochemical staining of TH was analyzed in SNpc and ST, respectively. Values were expressed as mean ± SEM (6-OHDA-lesioned group; n = 10, sham-operated group; n = 9). Scale bar = 100 μm. #p<0.05 and ###p<0.001 (vs. sham-operated group). (PDF) [file pone.0217194.s001.pdf]

### Principal coordinates analysis [UniFrac, Species, Include Unclassified OTUs (Reads)]

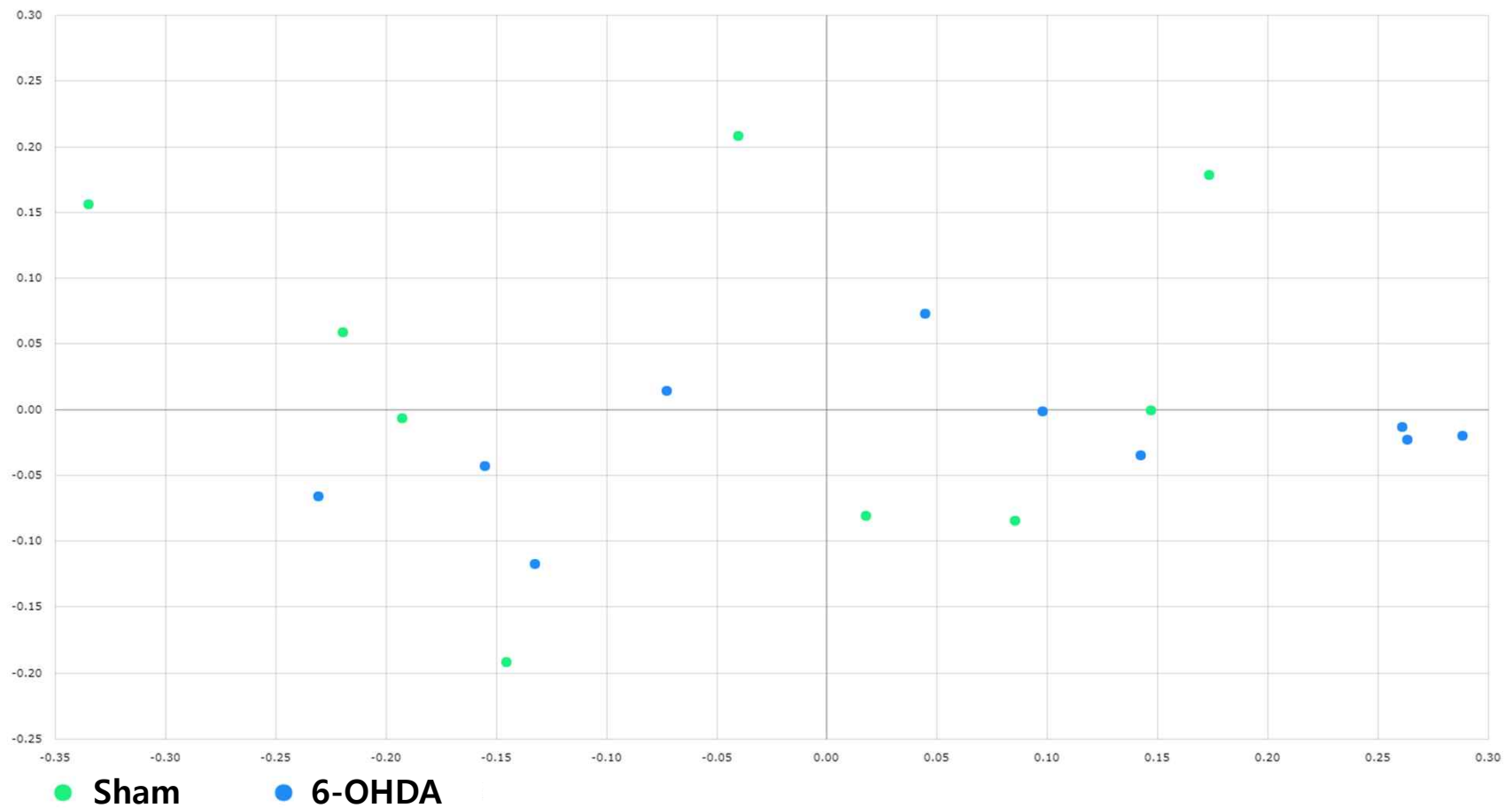

Supplement: S2 Fig — OTUs were determined based on 97% similarity of reads. Green and blue dots denote the sham and 6-OHDA group, respectively. (PDF) [file pone.0217194.s002.pdf]
