# Supplementary material for: High-throughput 16S rRNA gene sequencing reveals that 6-hydroxydopamine affects gut microbial environment
Source: PLoS One. 2019 Aug 12;14(8):e0217194. doi: 10.1371/journal.pone.0217194 (PMC6690581; doi:10.1371/journal.pone.0217194)
Supplement: S1 Text — (DOCX) [file pone.0217194.s003.docx]

**Methods**

**Apomorphine-induced rotation test**

The apomorphine-induced rotation test is the most commonly used behavioral test in unilateral lesion of striatum (1, 2). The rotation of all mice induced by apomorphine was tested at 14 d after the surgery. Both sham-operated and 6-OHDA-lesioned mice were placed in hemispheric rotational bowl with a diameter of 40 cm. They were allowed to habituate for 5 min before the administration of apomorphine (4 mg/kg, *s.c.*). The rotations in the direction opposite to the lesion were measured for 25 min.

**Rotarod test**

The rotarod test is a useful method for measuring hypokinesia in a mouse model of PD (3, 4). We performed the rotarod test at 14 d after 6-OHDA lesion. The rotarod unit consists of a rotating spindle (7.3 cm diameter) and five individual compartments able to simultaneously test five mice. After two successive days of twice-daily training (4 rpm rotation speed on the first day and 20 rpm on the second day), the test rotation speed was increased to 25 rpm on the third day in a test session. The time each mouse remained on the rotating bar was recorded over three trials per mouse, at 5 min intervals and a maximum trial length of 300 s per trial. Data are presented as mean time on the rotating bar over the three test trials.

**Open field test**

The open field test is a useful method to measure poor gait and ambulation in mice model of PD (3, 5). We performed the open field test at 14 d after 6-OHDA lesion. All measurements were made between 9 p.m. and 2 a.m. to avoid diurnal variation. The mice were placed in the testing chamber (40 × 25 × 18) with black floors for 15 min adaptation, followed by a 30-min recording period using a computerized automatic analysis system (Viewer; Biobserve, Bonn, Germany). The data collected by computer included the total distance (pixel) traveled by tracking the center of the animal.

**Immunohistochemistry**

At 14 d after 6-OHDA injection, mice were anesthetized with tribromoethanol (312.5 mg/kg, *i.p.*) and sacrificed by perfusion using 0.05 M PBS with 4% paraformaldehyde fixation. The brains were post-fixed overnight at 4℃ in the same solution and soaked in 0.05 M PBS containing 30% sucrose for cryoprotection. Frozen brains were cut into 30 μm coronal sections using a cryostat microtome. Free-floating sections were pre-incubated overnight at room temperature with a polyclonal rabbit anti-TH antibody (1:2000; Chemicon, Temecula, CA, USA). All sections were incubated with anti-rabbit secondary antibodies (1:200; Vector Labs, Burlingame, CA, USA) for 90 min, and with an ABC (Vector Labs, Burlingame, CA, USA) for 1 h at room temperature. Peroxidase activity was visualized by incubating sections with DAB for 3 min. Quantification of the number of dopaminergic neurons in brain sections was performed by counting the TH-immunopositive cell number in substantia nigra pars compacta (SNpc) at 100× magnification under a microscope (AxioSkop 2; Carl Zeiss Inc., Göttingen, Germany). The density of TH-positive fibers in ST was estimated as described previously (6).

**Statistical analysis**

All statistical parameters were calculated using GraphPad Prism 5.0 software (GraphPad Software Inc., San Diego, USA). Values were expressed as the mean ± standard error of the mean (SEM). The results were analyzed with the Student’s t-test between two groups. Differences with a *p* value less than 0.05 were deemed to be statistically significant.

**References**

1. Ungerstedt U, Arbuthnott GW. Quantitative recording of rotational behavior in rats after 6-hydroxy-dopamine lesions of the nigrostriatal dopamine system. Brain Res. 1970;24(3):485-93.

2. Iancu R, Mohapel P, Brundin P, Paul G. Behavioral characterization of a unilateral 6-OHDA-lesion model of Parkinson's disease in mice. Behav Brain Res. 2005;162(1):1-10.

3. Gu PS, Moon M, Choi JG, Oh MS. Mulberry fruit ameliorates Parkinson's-disease-related pathology by reducing alpha-synuclein and ubiquitin levels in a 1-methyl-4-phenyl-1,2,3,6-tetrahydropyridine/probenecid model. J Nutr Biochem. 2017;39:15-21.

4. Rozas G, Lopez-Martin E, Guerra MJ, Labandeira-Garcia JL. The overall rod performance test in the MPTP-treated-mouse model of Parkinsonism. J Neurosci Methods. 1998;83(2):165-75.

5. Schwarting RK, Sedelis M, Hofele K, Auburger GW, Huston JP. Strain-dependent recovery of open-field behavior and striatal dopamine deficiency in the mouse MPTP model of Parkinson's disease. Neurotox Res. 1999;1(1):41-56.

6. Moon M, Kim HG, Hwang L, Seo JH, Kim S, Hwang S, et al. Neuroprotective effect of ghrelin in the 1-methyl-4-phenyl-1,2,3,6-tetrahydropyridine mouse model of Parkinson's disease by blocking microglial activation. Neurotox Res. 2009;15(4):332-47.
