# Supplementary material for: High-throughput 16S rRNA gene sequencing reveals that 6-hydroxydopamine affects gut microbial environment
Source: PLoS One. 2019 Aug 12;14(8):e0217194. doi: 10.1371/journal.pone.0217194 (PMC6690581; doi:10.1371/journal.pone.0217194)
Supplement: S2 Text — (DOCX) [file pone.0217194.s004.docx]

**Methods**

**Statistical analysis**

The result of a principal coordinates analysis using the UniFrac tool was evaluated by Permutational multivariate analysis of variance.
